# Cost of Goods Analysis Facilitates an Integrated Approach to Identifying Alternative Synthesis Methodologies for Lower Cost Manufacturing of the COVID-19 Antiviral Molnupiravir

**DOI:** 10.12688/gatesopenres.13509.1

**Published:** 2022-02-16

**Authors:** Christopher Peterson, Sayan Paria, Anita Deshpande, Saeed Ahmad, Andrew Harmon, John Dillon, Trevor Laird

**Affiliations:** 1 Latham BioPharm Group, Elkridge, Maryland, 21705, USA; 2 Medicines for All Institute, VCU, Richmond, Virginia, 23298, USA; 3 JLD Pharma Consulting, LLC, Tinton Falls, New Jersey, 07724, USA; 4 Trevor Laird Associates Ltd, East Sussex, TN21 0TG, UK

**Keywords:** molnupiravir, uridine, cytidine, COVID-19, SARS-CoV-2, coronavirus, Cost of Goods

## Abstract

Orally delivered drugs offer significant benefits in the fight against viral infections, and cost-effective production is critical to their impact on pandemic response in low- and middle-income countries. One example, molnupiravir, a COVID-19 therapy developed by Emory, Ridgeback, and Merck & Co., had potential to benefit from significant cost of goods (COGs) reductions for its active pharmaceutical ingredient (API), including starting materials. A holistic approach to identifying, developing, and evaluating optimized synthetic routes, which includes detailed COGs modeling, provides a rapid means to increase the availability, uptake and application of molnupiravir and other antivirals in global markets.

Identification and development of alternate processes for the synthesis of molnupiravir has been conducted by the Medicines for All Institute at Virginia Commonwealth University (M4ALL) and the Green and Turner Labs at the University of Manchester. Both groups developed innovative processes based on synthetic route design and biocatalysis aimed at lowering costs and improving global access. The authors then performed COGs modeling to assess cost saving opportunities. This included a focus on manufacturing environments and facilities amenable to global public health and the identification of key parameters using sensitivity analyses.

While all of the evaluated routes provide efficiency benefits, the best options yielded 3-6 fold API COGs reductions leading to treatment COGs as low as <$3/regimen. Additionally, key starting materials and cost drivers were quantified to evaluate the robustness of the savings. Finally, COGs models can continue to inform the focus of future development efforts on the most promising routes for additional cost savings.

While the full price of a treatment course includes other factors, these alternative API synthetic approaches have significant potential to help facilitate broader access in low- and middle-income countries. As other promising therapeutics are developed, a similar process could enable rapid cost reductions while enhancing global access.

## Introduction

Molnupiravir (EIDD-2801, MK-4482) is an investigational, orally bioavailable prodrug shown to inhibit replication of severe acute respiratory syndrome-related coronavirus 2 (SARS-CoV 2). At the onset of the coronavirus disease 2019 (COVID-19) pandemic, Emory University initiated the development of molnupiravir for the treatment of SARS-CoV-2 infection. Further development was executed after licensing by Ridgeback Therapeutics and a partnership agreement with Merck and Co. Those efforts have led to promising results in recent phase 2/3 clinical trials. The United States recently approved the use of molnupiravir as a treatment option for COVID-19 via the Emergency Use Authorization Application.

Earlier in 2021, the U.S. government entered into a supply agreement with Ridgeback/Merck and has exercised multiple purchase options for approximately 3.1 million courses of the treatment upon EUA approval at a cost of $2.2 billion USD ^1^ of which Cost of Goods (COGs) is likely to be a small portion. On November 4, 2021, the UK Medicines Health Regulatory Authority (MHRA) authorized molnupiravir for the treatment of mild-to-moderate COVID-19 in adults who have tested positive for COVID-19 and at least one of the risk factors for developing serious illness. Advanced purchase agreements were entered for approximately 480,000 courses pending approval. Additional high-income countries such as Japan ^2^, Australia, South Korea, and Singapore ^3^ have secured agreements from 20 thousand to 1.6 million treatments pending the drug’s approval.

Nevertheless, when looking at the potential distribution of molnupiravir in low- and middle-income countries, COGs for the manufacturing of Active Pharmaceutical Ingredient (API) are likely to have a significant impact on the ultimate global accessibility. Fortunately, several potentially lower cost routes have been developed and are publicly available.

To assess the potential impact of these lower cost routes, we have modeled and compared five of the most efficient manufacturing routes published for molnupiravir and report herein a COGs analysis. To do this, we leveraged a small molecule cost modeling tool that was developed to account for the manufacturing strategies commonly used in more price-sensitive markets. The results allow generic manufacturers to evaluate potentially more cost-effective routes for producing molnupiravir and help drive the production of lower cost API for low- and middle-income countries.

### Small molecule API cost modeling

The use of cost models to investigate pharmaceutical COGs is common practice. We have collected comprehensive datasets and developed a model that overcomes many of the shortcomings of applying traditional models to pharmaceuticals made for highly price-sensitive markets. One key difference is the strategic use of excess capacity, as opposed to the design of a purpose-built facility or suite. Other differences include the costs associated with manufacturing in different geographies or for different regulatory authorities, and the cost of raw materials at different volumes for more segmented markets. Ultimately, the key to understanding these impacts is to model them and run sensitivities to highlight the key assumptions and output ranges.

It is important to note that COGs models, including this one, rarely account for all the costs that would go into determining price. There are many costs ranging from development costs to commercialization costs that would need to be accounted for when evaluating the margins that would result from a given pricing model which are not included in this COGs analysis. Nevertheless, COGs is a key element in the calculation, and the results produced by our models provide a strong starting point for this more detailed analysis.

### Synthesis of molnupiravir (EIDD-2801, MK-4482)

N ^4^-hydroxycytidine (NHC) was first synthesized in 1959 ^4^ by treating 4-thiouridine with hydroxylamine in ethanol. Over the following several decades, NHC was shown to be mutagenic to certain bacteria and viruses and, as early as 2004, was reported to inhibit SARS-CoV ^5^. A decade later, researchers at Emory University reported NHC as a broad-spectrum antiviral against influenza and respiratory syncytial viruses (RSV) ^6^ and subsequently developed the 5 isopropylester prodrug of NHC (EIDD-2801, known today as molnupiravir). COGs analysis of this original synthesis route was not performed in this study.

### Initial evaluation

A high-level route chemistry and manufacturing expert evaluation of this compound was performed based on patent applications around EIDD 2801 ^7, 8^ filed prior to its licensing to Ridgeback. This evaluation was not a COGs modeling effort as the full route is not publicly available, but the expectation was that there are several drivers which would cause the EIDD-2801 COGs to be higher *versus* the five modeled routes. Due to certain structural features, EIDD-2801 was a challenging molecule to synthesize. Specialized, expensive reagents were required, and the original starting material (uridine) was more expensive than cytidine. The patented route required five steps and utilizes chromatography at several steps to purify intermediates, which were not conducive to a low-cost, industrial route.

To address the anticipated issue of API cost, research groups including the Medicines for All Institute at Virginia Commonwealth University (M4ALL) and the Green and Turner Labs at the University of Manchester, UK approached the synthesis of molnupiravir systematically. Upon evaluation of the known routes, they developed alternative approaches that reduce API costs (e.g., yield improvement, manufacturing efficiency, and use of low-cost materials). They further explored leveraging biocatalysis to generate a two-step route. All route development is available via open-access publications including the custom biocatalysis outlined in the University of Manchester Biocatalysis Route developed by the Green and Turner Labs.

### Emory University, uridine route (5 steps, 17% yield)

**Figure 1.**
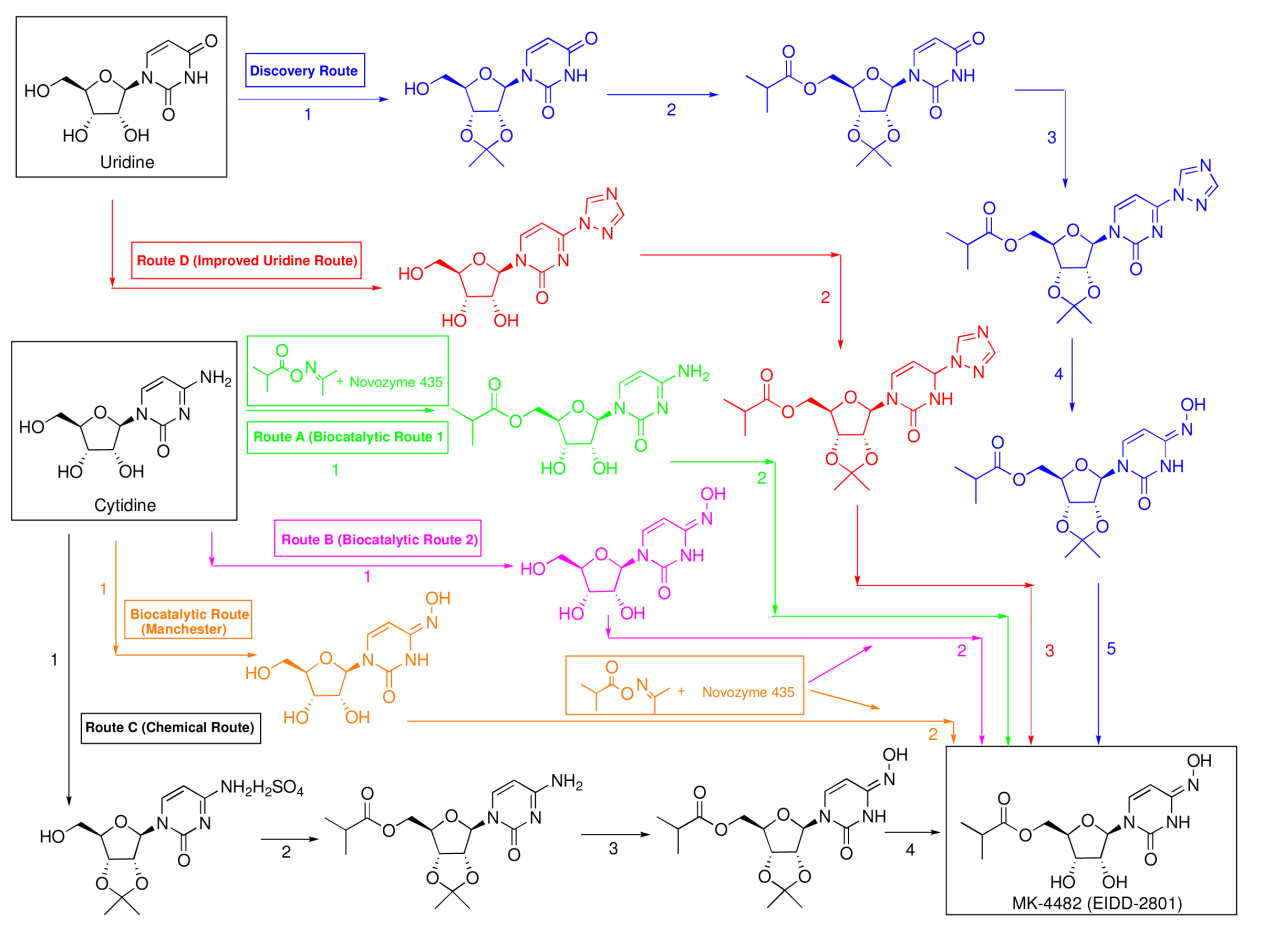
Modified uridine discovery route (D) and cytidine process routes (A, B, C, Manchester).

The initial synthesis of molnupiravir as reported by Emory University ^7, 8^ entailed a five-step procedure starting from uridine ( Figure 1, Discovery Route). The route required protection of the 2’- and 3’-hydroxy groups as an acetonide and esterification of the 5’-hydroxy group (99% yield over 2 steps) prior to coupling uridine with triazole at the 4-position (29% yield). Replacement of the resulting 4-triazolo group with hydroxylamine (60% yield) was followed by deprotection with formic acid (yield not reported) to give molnupiravir. A recrystallization step was also required to provide API of 99% purity.

This discovery route was considered inefficient for large scale manufacturing since it requires five steps and had a low overall yield (17%). In earlier work, Painter *et al.* ^7, 8^ described an alternative synthetic route starting from the less expensive cytidine (~$60/kg cytidine versus ~$160/kg uridine) in which the 4-amino group could be directly replaced with hydroxylamine in one step but required a pressure vessel and proceeded in only 25% after chromatographic purification.

### M4ALL improved uridine route (5 Steps, 61% yield)

M4ALL, in collaboration with Kappe’s group at Graz, Austria, improved the Painter route by reordering the five steps (triazole formation, protection, esterification, oximation, then deprotection), devising methodology to create two one-pot routes, and incorporating continuous flow into the last step to address the exothermic nature of the sulfuric acid mediated acetonide deprotection ( Figure 1, Route D) ^9^. In effect, this route reduced the number of steps and raw materials used and produced molnupiravir in 61% overall yield from uridine. By generating an improved version of the uridine route, manufacturers who are currently using uridine or that have stockpiled uridine will be able to generate molnupiravir with greater cost advantages when compared to the traditional route.

### M4ALL chemical route from cytidine (4 steps, 44% yield)

M4ALL devised an all-chemical (non-enzymatic) synthetic route starting from cytidine ( Figure 1, Route C) that involves 2’-,3’-protection using acetone, non-enzymatic acylation, oximation, and deprotection. This four-step route used low-cost reagents to produce molnupiravir in an overall 44% yield ^10^. Recent inquiry has shown that cytidine is available on the commercial marketplace in the multi-metric ton scale ^11^.

### M4ALL biocatalytic routes from cytidine (3 steps, 60% yield)

Researchers at Medicines for All Institute (M4ALL), in collaboration with the Massachusetts Institute of Technology (MIT) and University of Mainz, Germany, envisaged a two-step route involving oximation with hydroxylamine followed by esterification. Conversely, the synthesis could be achieved by esterification first followed by oximation. These routes eliminated the need to protect the 2’ and 3’ positions of cytidine ( Figure 1, Routes A and B) ^12^. Noteworthy, the synthesis of the esterification reagent required a separate step, so in practice, Routes A and B are considered three-step routes.

Both routes were optimized for manufacturing to improve yields and reduce the cost of goods. Route A (the process of enzymatic esterification followed by oximation/transamination) was further advanced in collaboration with Tim Jamison’s group at MIT and was shown to provide a 41% overall yield of molnupiravir from cytidine in two steps ^13^. The preferred route, Route B, was further refined at M4ALL to initially produce NHC as an easy-to-isolate crystalline intermediate. While the subsequent enzymatic acylation of NHC provided a mixture of 5’-OH acylation product and di-acylation (acylation on both the 5’-OH and 4-oxime groups) side product, it was found that treatment with hydroxylamine converted the latter by-product directly to molnupiravir. Overall, Route B produced molnupiravir in >99% purity in 60% yield from cytidine on a 100 g scale ^11^.

### University of Manchester biocatalytic route from cytidine (3 steps, 61% yield)

In parallel, the Green and Turner Labs at the University of Manchester, UK investigated the biocatalytic conversion of cytidine to NHC using an engineered variant of cytidine deaminase (Prozomics UK), that has been adapted by directed evolution to favor hydroxyaminolysis over hydrolysis ^14^. This novel enzyme operated at high substrate concentration (180 g/L) and provided a high yield (>90%) of NHC, which crystallizes directly from the reaction mixture ^14^. The enzyme was available from Prozomix at relatively low cost and is not restricted by IP. Subsequent enzymatic esterification of NHC was achieved following step 2 of Route B, Figure 1. The all-biocatalytic synthetic route is potentially scalable and may provide the lowest cost process for manufacturing of molnupiravir. Ongoing scale-up work and enzyme recycling optimization is being performed in concert with Sterling Pharmaceuticals and the Manchester Group.

Merck published a route starting from commodity chemicals, ribose and uracil, which are converted to molnupiravir in high yields in three biocatalytic steps; reactions use designer enzymes (Codexis) derived by directed evolution, and these enzymes are not currently commercially available to other manufacturers ^15^.

## Methods

Many parameters were studied to generate a COGs assessment for the various molnupiravir synthesis routes. Manufacturing was modeled at a benchmarked facility in India with a total of 60,000L reactor volume and meeting the requirements for Stringent Regulatory Authority (SRA) markets. The following costs were evaluated:

- Breakdown of time per individual process step
- Estimates of routine processing steps if unknown (i.e., material additions)
- Processing conditions for each step
- Material balance for scale-up and optimization
- Facility utilization
- Labor requirements

The Latham BioPharm Group model utilizes various public sources to determine labor and facility cost assumptions. These assumptions were validated and supplemented through interviews with local industry stakeholders and tailored specifically to the context of manufacturing in India. This allowed the cost of operations of a facility with 60,000L of available reactor capacity along with the additional, expected processing equipment to be estimated.

### Determining optimized API batch size assumptions

Kilo lab batch records and lab data associated with the published synthesis routes were used to estimate the process time and conditions. For any given process, the longest process step provides the batch-turnaround time for the facility. To correct for extreme conditions, defined as pressure > 200 psi or < atmospheric and/or temperatures > 200×C or < -10×C, published heuristics were used to upcharge various individual process steps towards the facility cost to simulate the additional equipment charges for such conditions ^16^.

Individual unit operations were also bucketed into stages which allowed for set tasks to be allocated by stage process yields ( *i.e*., raw materials converted to an intermediate at X%) instead of utilizing an overall, total process yield.

The kilo lab or bench scale data was assessed for potential optimization opportunities ( *e.g*., lack of optimal tank usage during bench scale and kilo lab scale). This allowed the identification of the optimal batch size at the given scale of the data. Once this value was achieved, scaling was performed linearly to calculate the amount of material that could be achieved in our modeled 60,000 L facility with the assumptions that lab yields could be replicated at manufacturing scale. An annual demand of 50 metric tons was used to generate a yearly facility utilization based on the calculated process time (batch-turnaround) as well as the associated raw material requirements.

This model assesses the costs to manufacture in excess capacity. Additionally, it only assesses the API facility costs on a utilization basis. There are fixed costs for batch turnaround time, but additional facility costs are embedded within facility utilization ( *e.g*., if 1.5× facilities are needed then facility utilization would equal 150%).

### Material balance assumptions

Material balance is the amount of material used in a particular step of the lab/kilo lab/manufacturing process. The material balance was derived for each stage in a particular process. Input and output materials were categorized and quantified at the lab/kilo lab scale. The model tracked the material balance on a molar or mass basis, and the output material was calculated based on real-world yields.

Raw material costs were obtained through direct quotes, proprietary databases ( *e.g.*, import/export), or public information. The costs were adjusted for the predetermined scale. These costs were standardized across the various routes. As all raw materials are subject to price fluctuations over time, and material cost assumptions are specific to information gathered at a point-in-time, sensitivity analyses were used to demonstrate the impact of price fluctuations.

The materials were categorized in the model as catalysts, solvents, enzymes, or intermediates. Catalysts and solvents were assumed to be recycled at rates of 90% and 75%, respectively (the molnupiravir routes in this document do not utilize catalysts). In this model, enzymes have both a recycle / top-off (per batch) and a replacement factor after a pre-determined number of uses (# of batches). Routes which used an enzyme were standardized to four uses with a top-off of 10% for every non-‘fresh’ batch.

After the material balance was generated for the provided lab/kilo lab scale, all raw materials were scaled up to the assumed annual volume (50 metric tons). This ensured correct calculations compared to the real-world data.

The top five most expensive raw materials were automatically identified to allow for sensitivity analysis for potential pricing changes in various scenarios. Additionally, process yields were assessed at each stage to identify the stages in which process yield improvements would have the highest impact on COGs.

### Assumptions for conversion of API to final dosage form

Industry experts were engaged to validate API conversion cost estimates to final dosage form. These per dose estimates were then assessed on a treatment regimen basis of 800 mg of API dosed 2× daily over 5 days for molnupiravir. Functionality to assess the cost of formulating the API into finished pharmaceutical product was included in the model. After converting the API costs to a per-dose value, the model added raw material and conversion costs to calculate a total COGs per regimen ( Table 1).

**Table 1.** Variables and associated costs for the final dosage form of molnupiravir.

Variable	Range
Facility capacity	65 million – 1.5 billion doses
Excipient and capsule costs	$0.01–$0.03 / dose
API conversion (inclusive of packaging)	$0.025–$0.10 / dose

## Results and discussion

For the COGs analysis, five synthetic routes were modeled using the methodology above:

**1.**  **M4ALL improved uridine route – Route D ( Figure 1)**
**2.**  **M4ALL chemical route from cytidine – Route C ( Figure 1)**
**3.**  **M4ALL biocatalytic route A from cytidine – Route A ( Figure 1)**
**4.**  **M4ALL biocatalytic route B from cytidine – Route B ( Figure 1)**
**5.**  **University of Manchester biocatalytic route from cytidine – Biocatalytic Route ( Figure 1)**

### 1. M4ALL Improved Uridine Route (Route D)

The first route that was assessed was for the improved synthesis of molnupiravir from uridine by M4ALL. The process reduced the number of steps from five to three. The model estimated the total COGs for this route to be $467/kg. Cost benefits in the route were achieved by telescoping key parts of the 5-step synthesis into 3 steps, increasing solvent recycling, and decreasing time in plant/facility costs.

**Figure 2.**
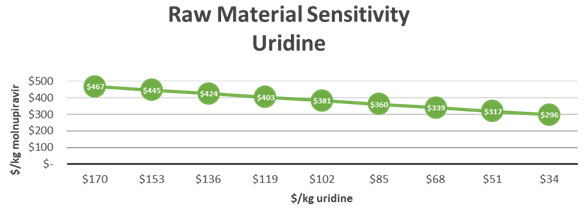
M4ALL Improved Uridine Route sensitivity analysis for Uridine.

**Figure 3.**
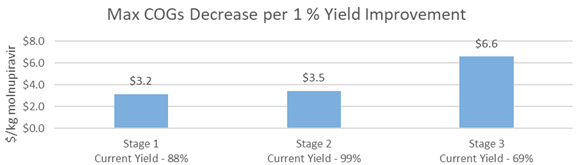
M4ALL Improved Uridine Route maximum COGs decrease per 1% yield improvement in each step.

API cost estimates are sensitive to the cost of raw materials. In addition, the API cost estimates are sensitive to the changes in the yield. In this example, the cost of molnupiravir was sensitive to the cost of uridine and hydroxylamine. The price of uridine was estimated at $170/kg resulting in a modeled API cost estimate of $355,834 per batch (~1.7 MT) of molnupiravir. To visualize the sensitivity of molnupiravir API cost estimates to uridine, a decreasing cost of uridine was modeled as seen in Figure 2. Decreasing the cost of uridine by 10% led to a $21 decrease in molnupiravir API costs/kg. The third step of the reaction coupled hydroxyamination and acetonide deprotection to form molnupiravir from an acetonide ester intermediate with a 69% yield. A 1% yield improvement in this step led to a $7 decrease in cost/kg ( Figure 3). If uridine remains highly available and prices trend downward, manufacturers can also benefit from overall API cost reductions.

### 2. M4ALL chemical route from cytidine (Route C)

**Figure 4.**
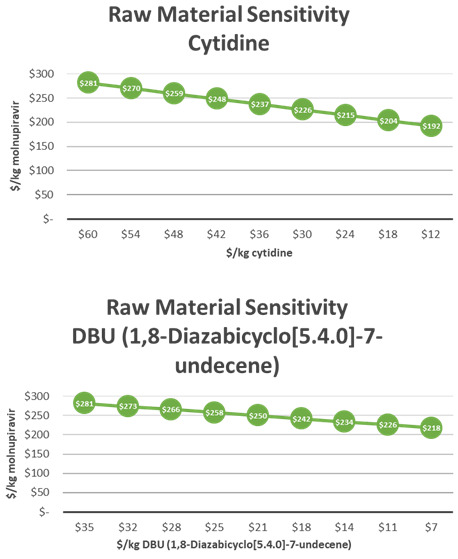
M4ALL Chemical Route from Cytidine sensitivity analysis for (a) cytidine and (b) 1,8-Diazabicyclo[5.4.0]-7-undecene.

In the M4ALL chemical route from cytidine (Route C, Figure 1), molnupiravir was synthesized using cytidine as a starting material in a four-step process. The modeled cost of molnupiravir from this route was $281/kg. The price of cytidine was currently stable at $60/kg and was significantly more cost-effective as a starting material than uridine. To visualize the sensitivity of the molnupiravir API cost estimates to the cytidine starting material, a decreasing cost of cytidine was modeled as seen in Figure 4. Decreasing the cost of cytidine by 10% led to an $11/kg decrease in molnupiravir. Another key cost driver in this synthesis was 1,8-diazabicyclo[5.4.0]-7-undecene (DBU). DBU was used as a base in the second step of the reaction. An estimate price of $35/kg DBU was used in the model. The sensitivity of molnupiravir API COGs was assessed for changes in the yield of this second step. A 10% decrease in price of DBU led to a modeled decrease in the COGs of molnupiravir by $8/kg.

**Figure 5.**
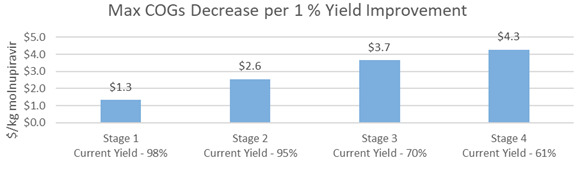
M4ALL Chemical Route from Cytidine maximum COGs decrease per 1% yield improvement in each step.

There is an opportunity for manufacturers to further optimize step 3 (70% yield) and step 4 (61% yield). Improvements in either of these two steps is modeled to reduce the COGs of molnupiravir API of $3.7/kg and $4.3/kg, respectively, for every 1% increase in yield. ( Figure 5).

### 3. M4ALL biocatalytic route A from cytidine (Route A)

As noted in Figure 1, the enzyme Novozyme 435 (a proprietary version of *Candida antarctica* lipase B – CALB, fixed on resin beads) was used in the first step of this 2-step process. Each of the biocatalytic routes was sensitive to the price of Novozyme 435. Using vendor quotes, Novozyme 435 was priced at $1,200/kg. The oxime ester coupling with the 5’hydroxyl group was an irreversible reaction as it produced acetone and hydroxylamine driving isobutyrylation via Novozyme 435. Utilizing the most expensive material in the first step of the process magnified its impact on API COGs. The modeled estimate of the molnupiravir API COGs for this route was $1,274/kg with default enzyme recycling. In addition, this route required an extended reaction time of 40+ hour at 100ºC (step 2) and provided a lower reaction yield leading to double facility utilization. Given that other successful routes to molnupiravir were able to utilize Novozyme 435 in later steps, this process is unlikely to be adopted by manufacturers.

### 4. M4ALL biocatalytic route B from cytidine (Route B)

M4ALL biocatalytic route B from cytidine (Route B, Figure 1) reversed the steps from the previous route and utilized Novozyme 435 in the final step with oxime ester to convert N-hydroxycytidine (NHC) hydrate to molnupiravir. By utilizing Novozyme 435 later in the synthesis the API costs significantly improved. The total COGs for this reaction were modeled at $205/kg with 9% of COGs attributed to labor and facility costs.

**Figure 6.**
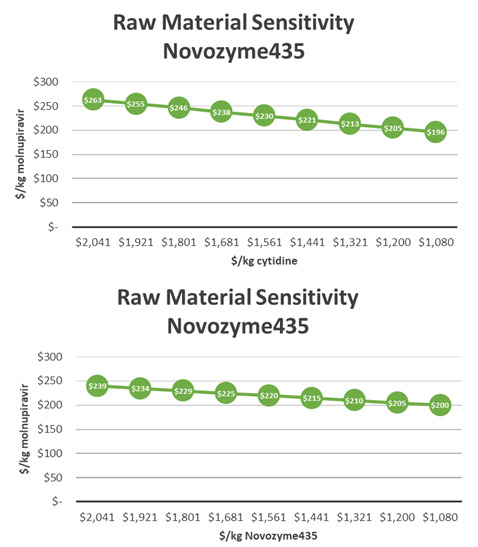
M4ALL Biocatalytic Route B from Cytidine sensitivity analysis for (a) cytidine and (b) Novozyme 435.

**Figure 7.**
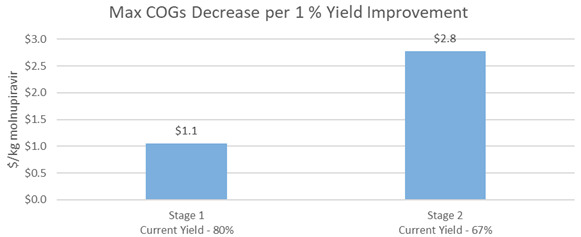
M4ALL Biocatalytic Route B maximum COGs decrease per 1% yield improvement per step.

**Figure 8.**
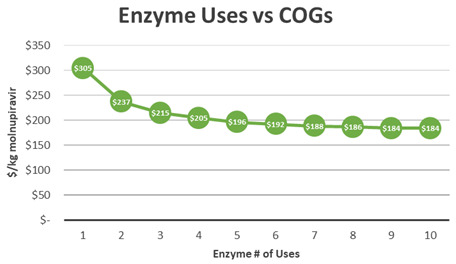
M4ALL Biocatalytic Route B from Cytidine Enzyme usage versus COGs.

The key cost drivers in this synthesis were Novozyme 435 and cytidine ( Figure 6). A 10% decrease in the price of the Novozyme 435 led to a $5/kg decrease and a 10% decrease in the price of cytidine led to a $8.3/kg decrease in molnupiravir API COGs. Increasing the yield of stage 2 by 1% would result in a $1/kg decrease in COGs ( Figure 7). It is critical to note that the cost of this process can be significantly improved by recycling Novozyme 435. The process has been demonstrated at kilogram scale with up to three recycles, topping off with 10% fresh enzyme after each batch. An initial recycling of enzyme leads to a reduction of molnupiravir COGs by $67/kg. As recycling increases, the effect of cost reduction decreases. After 10 recycles, minimal reduction in molnupiravir COGs is achieved ( Figure 8).

### 5. University of Manchester biocatalytic route from cytidine

**Figure 9.**
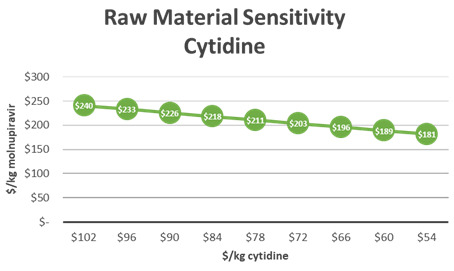
Biocatalytic Route (Manchester) raw material sensitivity for Cytidine.

**Figure 10.**
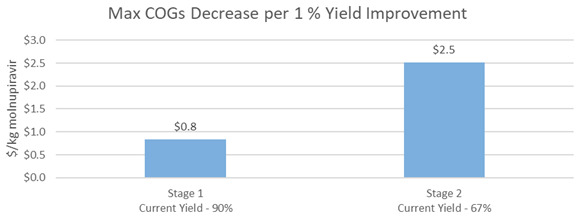
Biocatalytic Route (Manchester) maximum COGs decrease per 1% yield improvement for Biocatalytic Route.

**Figure 11.**
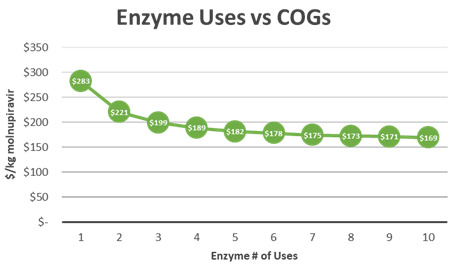
Biocatalytic Route (Manchester) – Enzyme Use versus COGs.

The Manchester group invented a biocatalytic method to convert cytidine to NHC, replacing the first chemical step in Route B with an enzymatic conversion, but utilized less harsh process conditions and generated less waste. Using a custom enzyme, they increased the yield of the first step by 9%, which increased the overall yield of the reaction. The modification decreased total API COGs to $189/kg ( Figure 9, Figure 10). Additionally, the Manchester Group is working with Sterling Pharmaceuticals to optimize the scale up of their Biocatalytic route as well as optimize the enzyme recycling routes at scale. The recycling process modeled in this paper results in diminishing returns after the 4 ^th^ use of the enzyme ( Figure 11). It is critical to note that this is only one methodology of recycling, and that commercial scale recycling could result in different economics depending on the capabilities of manufacturers to optimize this process at their facilities.

### 6. API and regimen COGs outputs

Table 2 illustrates the outputs and associated costs of all five synthetic routes of molnupiravir manufacture examined as part of the COGs analysis as well as estimates of the conversion of API into final dosage form allowing for a comparison on a regimen basis.

**Table 2.** API and Regimen COGs outputs and associated costs of synthetic routes of molnupiravir manufacture.

Outputs	Improved Uridine Route	Cytidine Chemical Route	Biocatalytic Route A from Cytidine *	Biocatalytic Route B from Cytidine *	University of Manchester Biocatalytic Route *
	$/kg	$/kg	$/kg	$/kg	$/kg
Labor:	$2	$7	$79	$7	$7
Raw Materials:	$461	$254	$1,067	$185	$170
Facility:	$4	$21	$128	$13	$12
**Total API COGs:**	**$467**	**$281**	**$1,274**	**$205**	**$189**
					
COGs/Regimen **:	$5.0	$3.6	$11.5	$2.9	$2.8

*Default enzyme recycling (4 uses, 10% top-off) **Includes final dosage form costs

## Conclusions

The COGs for five publicly available, potentially lower cost molnupiravir routes were modeled that utilize alternative manufacturing methodologies from a combination of biocatalytic, chemical, and/or alternative starting materials (cytidine *versus* uridine). The model presents a comprehensive approach and considers key nuances of manufacturing for the lower-margin markets, including implementation as a function of fit based on an entrant’s capabilities and infrastructure. Results of the analysis suggest that the API COGs reduction development efforts could enable an overall reduction of 3–6-fold to as low as <$200/kg. This translates to final dosage form COGs/regimen ranging from < $3 to ~ $5. These results are sensitive to the starting material pricing and could rise or fall dependent on changes in that dimension. In the future, further API COGs reductions could potentially be achieved with additional process yield optimization.

While the calculated reductions do not translate directly to pricing, they provide key information for the evaluation of molnupiravir’s commercial viability for generics manufacturers and support successful and simultaneous introduction in low-, middle-, and high-income settings.

In addition to demonstrating the value of COGs modeling as an integral part of the process, the molnupiravir case study presents an important new paradigm and best practice in the development of new global health therapeutics. In contrast to numerous previous examples of new and highly efficacious global health treatments that had to wait years, sometimes decades, to be cost-optimized and then introduced in low- and middle-income settings, molnupiravir was optimized for cost and manufacturability in parallel with the studies to demonstrate clinical efficacy and safety. As a result, molnupiravir can be successfully introduced into low- and middle-income country settings in the same time frame as it is introduced in higher income settings, at a price that supports widespread access. The optimization of COGs through this simultaneous approach, rather than the typical innovator and then (sometimes years later) generic approach, could represent a new paradigm for high potential global health therapeutics in the future.

## Data availability

### Underlying data

All data underlying the results are available as part of the article and no additional source data are required.

### Reporting guidelines

CHEERS checklist for “Cost of Goods Analysis Facilitates an Integrated Approach to Identifying Alternative Synthesis Methodologies for Lower Cost Manufacturing of the COVID-19 Antiviral Molnupiravir”. https://doi.org/10.17605/OSF.IO/9C6PY

Data are available under the terms of the Creative Commons Attribution 4.0 International license (CC-BY 4.0).

## Consent

Not applicable. Study was based on aggregated data available from the literature.
