# Supplementary material for: Cost of Goods Analysis Facilitates an Integrated Approach to Identifying Alternative Synthesis Methodologies for Lower Cost Manufacturing of the COVID-19 Antiviral Molnupiravir
Source: Gates Open Res. 2022 Feb 16;6:8. [Version 1] doi: 10.12688/gatesopenres.13509.1 (PMC8901586; doi:10.12688/gatesopenres.13509.1)
Supplement: Peer review history

# Peer review: Reviewer response for version 1

**DOI:** 10.21956/gatesopenres.14772.r31768

**Published:** 2022-03-17

**Authors:** Francisco Alvarez

**Affiliations:** 1 Alvarez CMC Group, LLC, Southern California, CA, USA

This is an interesting article that compares different chemistries and their overall impact on the estimated final dollar per kg cost of mulnipiravir (summarized in Table 2). The authors have developed a model that incorporates multiple factors impacting cost (e.g. number of steps, process conditions, time, yields, facility usage, labor, etc). The information about the model or algorithm is presented qualitatively, but the arguments and logic behind the factors considered is sound and understandable. Since the goal of the paper is not to provide an actual cost, but a rough, but reasonable, estimate based on the process information described in the literature (nature of raw materials, solvents, number of steps, reaction conditions, yields, etc), as well as estimated costs related to labor, facilities, etc., it is important to note the very close correlation between raw material costs and the final estimated mulnipiravir cost (Table 2. With raw materials generally accounting for ~90% of the total mulnipiravir cost. The biocatalytic route A is slightly off compared to the other processes, with a higher labor and facility cost, but some of the reasons are explained by the authors (long reaction time with a high process temperature)).

 In this regard, the differences between the biocatalytic routes A and B are quite dramatic, since it is not just the number of steps that influence the final value, but the order on how they are introduced into the process. Given that these two routes use the same biocatalyst (Novozyme 435), it is not totally clear why the large difference. It may have been useful giving a more detailed breakdown of costs per stage, with yields and other related information to better understand the differences among these 2 routes.

 A related question from the information in Table 2, although clearly not the main focus of the paper, is to comment on when the facility and labor costs start to become more relevant, i.e. greater influence on the final mulnipiravir cost. It would have been useful to include in this table the overall facility time to produce a given quantity, or, alternatively, the estimated batch-turnaround time and estimated number of worker-hours per batch.

 The authors also recognize that raw material costs could fluctuate for multiple reasons. Therefore, the sensitivity analysis helps to provide valuable information as to what aspects of the process may require focusing on to obtain the greatest cost reduction. As noted, yield is a significant factor as it directly impacts raw material quantities (i.e. cost). 

 It is not clear from the figures in the paper what is being represented in the x-axis of the sensitivity analysis for the different raw materials. Note that in discussion for Route C, the authors indicate that the cost of cytidine is “currently stable at $60/kg", the related Figure 4 for the sensitivity analysis starts with this value and varies to show its impact on mulnipiravir $/kg cost.  However, the related analysis for Routes B (Figure 6) and Biocatalytic Route (Manchester) (Figure 9) have very different cost/kg values for cytidine.  Similalry, it is not clear why the base cost for mulnipiravir changes depending on which raw material is used for the sensitivity analysis (Figure 6: $263 starting cost with cytidine and $239 starting cost for Novozyme 435). 

 Finally, although not the focus of the paper, it would have been valuable to include general comments about the environmental impact of the processes being considered here. These may include another cost factor that will likely start to take greater significance across the globe.

Is the work clearly and accurately presented and does it cite the current literature?

Yes

If applicable, is the statistical analysis and its interpretation appropriate?

Not applicable

Are all the source data underlying the results available to ensure full reproducibility?

No source data required

Is the study design appropriate and is the work technically sound?

Yes

Are the conclusions drawn adequately supported by the results?

Yes

Are sufficient details of methods and analysis provided to allow replication by others?

Partly

Reviewer Expertise:

Pharmaceutical drug product development

I confirm that I have read this submission and believe that I have an appropriate level of expertise to confirm that it is of an acceptable scientific standard.

# Peer review: Reviewer response for version 1

**DOI:** 10.21956/gatesopenres.14772.r31766

**Published:** 2022-03-07

**Authors:** Shankar Suryanarayanan

**Affiliations:** 1 Operations Transformation LLC, Chicago, IL, USA

This cost modeling tool is unique in its ability to evaluate total cost impact of the manufacturing process, raw materials, manufacturing location and destination markets. The ‘what if's’ inform manufacturing decisions that impact scalability, cost of goods and time to market.

 Presumably, given the proprietary nature of the cost model and the voluminous amounts of data processed, detailed calculations have not been shared. However, experts from multiple organizations have collaborated and validated model outputs. The results are directionally correct and have been supported by highlighting key contributing factors. Besides the batch processes evaluated, where feasible, advanced manufacturing processes such as continuous flow chemistry should also be explored.

 For an innovative pharmaceutical product, cost of goods declines steadily from commercial launch in developed markets to patent expiry several years later. In the case of molnupiravir, because of the simultaneous launch as a patent protected product in developed markets and as a generic in low and middle income countries, cost reduction efforts had to be accelerated. As the paper demonstrates, modeling tools helped with these efforts. This project also demonstrates the important role global health organizations play by funding and publishing cost reduction opportunities that can steepen the learning curve to improve affordability and accessibility.

Is the work clearly and accurately presented and does it cite the current literature?

Yes

If applicable, is the statistical analysis and its interpretation appropriate?

Not applicable

Are all the source data underlying the results available to ensure full reproducibility?

Partly

Is the study design appropriate and is the work technically sound?

Yes

Are the conclusions drawn adequately supported by the results?

Yes

Are sufficient details of methods and analysis provided to allow replication by others?

Yes

Reviewer Expertise:

Global manufacturing and supply chain management in the life science industry.

I confirm that I have read this submission and believe that I have an appropriate level of expertise to confirm that it is of an acceptable scientific standard.

# Peer review: Reviewer response for version 1

**DOI:** 10.21956/gatesopenres.14772.r31769

**Published:** 2022-03-02

**Authors:** Ping Chang

**Affiliations:** 1 Adtech Pharma, Inc., Warwick, RI, USA

The article gives a timely comparison of novel approaches to synthesize molnupiravir, a potentially essential medicine to treat COVID-19 patients. Novel synthesis routes developed at Medicine for All Institute and elsewhere are described and assessed with the cost model. Although the cost model assumes commercial production in India, the cost comparison on the same basis differentiates the novel processes from the literature process fairly. Further cost analysis could be helpful in decision making regarding the novel processes, for example, cost sensitivity of production scale (batch size and annual volume). A manufacturer may not outright commit 60,000 liter reactors and 50 tons a year output, likely a dedicated facility. The cost of technology transfer and scale up would be taken into consideration in initial small scales, as a part of investment outlay. Overall, the article gives an encouraging assessment of manufacturing the important drug molnupiravir.

Is the work clearly and accurately presented and does it cite the current literature?

Yes

If applicable, is the statistical analysis and its interpretation appropriate?

Not applicable

Are all the source data underlying the results available to ensure full reproducibility?

Yes

Is the study design appropriate and is the work technically sound?

Yes

Are the conclusions drawn adequately supported by the results?

Yes

Are sufficient details of methods and analysis provided to allow replication by others?

Yes

Reviewer Expertise:

Active pharmaceutical ingredient, discovery development commercialization

I confirm that I have read this submission and believe that I have an appropriate level of expertise to confirm that it is of an acceptable scientific standard.
